# Laparoscopic versus Open Ovariectomy in Bitches: Changes in Cardiorespiratory Values, Blood Parameters, and Sevoflurane Requirements Associated with the Surgical Technique

**DOI:** 10.3390/ani12111438

**Published:** 2022-06-02

**Authors:** Silvia Fernández-Martín, Victoria Valiño-Cultelli, Antonio González-Cantalapiedra

**Affiliations:** 1 Rof-Codina Veterinary Teaching Hospital, Faculty of Veterinary, Universidade de Santiago de Compostela, 27002 Lugo, Spain; antonio.cantalapiedra@usc.es; 2 Department of Anatomy, Animal Production and Veterinary Clinical Sciences, Faculty of Veterinary, Universidade de Santiago de Compostela, 27002 Lugo, Spain; victoria.cultelli@usc.es

**Keywords:** dog, intra-abdominal pressure, laparoscopy, laparotomy, mechanical ventilation, neutering, ovariectomy, pneumoperitoneum, sevoflurane

## Abstract

**Simple Summary:**

Laparoscopic neutering techniques are widely used in veterinary medicine due to their numerous advantages compared to open ones. However, laparoscopy requires the creation of pneumoperitoneum to establish a working space, and it has been related to the occurrence of pathophysiological alterations. The objective of this study was to evaluate the potential global systemic changes associated to the establishment of the pneumoperitoneum in laparoscopic ovariectomy and to compare them with those determined in conventional open surgery in dogs. Additionally, we evaluated the gas anesthetic requirements based on the surgical technique. The results of our study suggested that the cardiorespiratory alterations related to pneumoperitoneum seemed to be well-tolerated and reversible after CO_2_ deflation. Regarding the anesthetic gas evaluation, we determined higher requirements during ovarian resection in the open ovariectomy group. In conclusion, laparoscopic neutering procedures should be currently recommended by veterinarians due to their lower invasiveness and resulting complications. Nevertheless, a thorough understanding of the changes associated with pneumoperitoneum is essential to plan and obtain an adequate perioperative management.

**Abstract:**

The aim of this study was to examine the cardiorespiratory and blood changes associated with pneumoperitoneum (PNP) in laparoscopic ovariectomy (LAP Ove), as well as sevoflurane requirements, comparing them to those determined in open surgery (LPT Ove). The study was performed in 16 bitches submitted to LAP or LPT Ove. The cardiorespiratory and end-tidal sevoflurane concentration values were recorded as follows: at the beginning of surgery (T1), after the right ovary resection (T2), after the left ovary resection (T3), and after surgical closure (T4). Blood samples were taken before and after PNP. Among the cardiorespiratory parameters, no differences were observed in the values of end-tidal CO_2_, minute volume, and heart rate. In the LAP Ove group, a significant increase in inspiratory pressures and a decreased compliance were identified at T2 and T3. Significant higher arterial pressure values were observed in both groups at T2 and T3, with this increase especially marked at T2 in the LPT Ove group. Sevoflurane requirements were significantly higher in the LPT group during ovarian resection. Finally, in terms of the hematochemical parameters, statistical differences were recorded between pre- and post-operative assessments, but not between both surgical groups. The pathophysiological effects associated with PNP seemed to be transient and well-tolerated by healthy dogs.

## 1. Introduction

In the last 15 years, minimally invasive surgery has shown an increasing success rate in veterinary medicine, and numerous techniques are being proposed for many surgical procedures. Among them, ovariectomy (OVE) and ovariohysterectomy (OHE) are some of the most common surgical techniques performed in small animals [1,2,3,4,5,6,7,8]. When evaluating the main advantages of laparoscopic neutering procedures compared to the open conventional ones, most of the authors highlight lower trauma associated with smaller incisions, as well as a lower number of postoperative complications [1,3,9]. Additionally, they also note lower rates of bleeding during surgery, less perioperative pain, reduced infection rates, decreased morbidity, and hospital stays [3,10,11,12].

Although laparoscopic surgery has many advantages, some complications have been described, mainly associated with the surgical access, the anesthesia management, and other procedures specific to laparoscopic techniques [13,14,15]. Pathophysiological alterations have also been related to the abdomen insufflation with CO_2_, which results in the establishment of pneumoperitoneum (PNP) [16]. Specifically, the PNP leads to an elevated intra-abdominal pressure (IAP) with the consequent cranial displacement of the diaphragm, which induces changes in ventilatory and cardiovascular dynamics, such as decreased venous return, increased peripheral vascular resistance, decreased cardiac output, increased resistance to thoracic expansion, and decreased lung volume [17,18,19]. The compression of the pulmonary parenchyma has been related to the appearance of atelectasis and ventilation/perfusion alterations [17,20]. Another critical event is associated with the insufflation of CO_2_ and its absorption through the abdominal cavity, which may produce acid-base and cardiorespiratory disturbances [21]. Presently, it is well-known that the severity of these alterations is positively correlated with higher IAP pressures [22,23]. For that reason, the current veterinary literature supports the use of IAP of maximum 10 mmHg in dogs [24,25] and below 6–8 mmHg in cats [26,27,28]. Additionally, the duration of the PNP also appears to be another limiting factor in laparoscopic surgery, having an impact directly proportional to the systemic absorption of CO_2_ and, therefore, increasing the number of complications associated with this fact [21,27,29]. However, even following the recommended pressure values, complications may occur [26,27].

In relation to the pathophysiological changes that may occur during the laparoscopic surgery both in human [19,20,30,31,32] and in veterinary medicine [17,21,24,33,34], numerous publications have thoroughly examined these potential alterations. Some studies on animals recorded effects in terms of cardiovascular [24,29,33,35], respiratory [17,25,26,27,34,36] and metabolic parameters [21,29,37].

On the other hand, several authors have examined the numerous advantages of minimally techniques compared to the open traditional ones in small animals. At present, most of them focus on the comparison of the perioperative complications, surgical recovery, and postoperative pain and morbidity [1,3,9,10,11,38], as well as inflammation response [18,39,40]. However, there are only a few publications evaluating the systemic effects during PNP in the LAP neutering procedures compared to the open techniques. Höglund et al. [41] recorded the changes in the blood pressure and heart rate during two surgical methods for neutering female dogs. Shariati et al. [10] examined perioperative complications, blood loss, as well as some clinical and blood parameters in laparoscopic and open surgery for OVE in dogs. Kumari et al. [42] evaluated the hemato-biochemical profile in bitches undergoing LAP and open Ove. Additionally, in another study on cats [27], the authors evaluated the cardiopulmonary effects of low IAP in LAP OVE comparing these findings to those obtained in an open surgery approach.

Nevertheless, to the best of the authors’ knowledge, there are no studies on dogs that have examined the potential global pathophysiological differences and the gas inhalation requirements between both OVE surgical techniques. Therefore, the aim of this study is to quantitatively evaluate the potential cardiorespiratory and blood changes associated with the establishment of PNP in laparoscopic OVE, and to compare them to those determined in conventional open surgery in dogs in a randomized, prospective clinical trial. For those purposes, we collected cardiovascular and ventilatory variables and performed gasometrical, electrolyte, biochemical, and hematological analyses during the surgical procedure. Additionally, we examined the requirements for minimum alveolar concentration (MAC) gas inhaled, depending on the surgical technique, using the end-tidal sevoflurane concentration (F_E’_Sevo, %) during anesthesia. We hypothesized that the pathophysiological changes related to PNP would be transient and well-tolerated by the dogs. Furthermore, we also hypothesized that open OVE would result in a high MAC requirements for sevoflurane compared to laparoscopic surgery.

## 2. Materials and Methods

### 2.1. Animals

This prospective, randomized clinical study was conducted in the Rof Codina University Veterinary Hospital (Lugo, Spain) and was approved by the Ethics Committee of the Rof Codina Galician Public Foundation.

The inclusion criteria for this study were healthy client-owned female dogs admitted for neutering procedure. All animal owners were informed about the detailed study protocols and a written informed consent was obtained for each dog from the pet owner. The included animals were randomly assigned into two groups depending on the surgical technique, according to a computer-generated randomization list: laparoscopic ovariectomy group (LAP Ove, *n* = 8) and laparotomy ovariectomy group (LPT Ove, *n* = 8). All the procedures were performed by the same veterinary surgical team.

All the animals underwent an anamnesis, a physical examination and a complete blood cell analysis, and a serum biochemistry profile. Therefore, only dogs assigned to the ASA I (American Society of Anesthesiologist) and weighing more than 15 kg were included. Dogs were excluded from the study if they were affected by systemic disease, pregnancy, cardiac arrhythmias, or any technical or anesthetic complications. Additionally, in order to determine the sevoflurane requirements depending on the surgical technique, we also decided to exclude the animals that need intraoperative rescue analgesia. The study included general data on breed, age, weight, and body condition. For body condition assessment, a scale of one to five was used, with one being thin and five being obese [43].

### 2.2. Equipment

The experimental work was carried out in the surgical area of the Rof Codina University Veterinary Hospital. The laparoscopic ovariectomies were performed using a Karl Storz^®^ endoscopy tower, consisting of modular equipment, which included: a Telecam camera with zoom, focus, brightness, and white balance control, a high-performance insufflator (Endoflator^®^ 50), a medical CO_2_ bottle, and a power LED light source with fiber optic light cable. Two 6 mm threaded trocars with a blunt fixator were used, with one of them provided with gas inlet, and one 11 mm threaded trocar also provided with a gas inlet and a rubber reducer to allow the use of 5 mm instruments. A Hopkins^®^ model optic of 30°, with 5 mm in diameter and 29 cm in length, from Karl Storz was also used. A 5 mm diameter curved tip dissector was used for handling and holding the ovary. The cutting and coagulation of the ovaries was performed using an Ultracision^®^ harmonic scalpel with advanced hemostasis (HARMONIC ACE^®^ + 7 handpiece, ETHICON) [11,44].

### 2.3. Anesthesia and Analgesia

All animals underwent general anesthesia after premedication with medetomidine (10 µg/kg/IM, Domtor, Esteve, Barcelona, Spain) and morphine (0.3 mg/Kg/IM, Morfina Braun 2%, B. Braun Medical, Barcelona, Spain). Approximately 15 min afterwards, and once the patient was mildly-to-moderately sedated, a cephalic vein was catheterized (Abbocath T-20G) and an IV infusion rate with Ringer–Lactate solution was started (5 mL/kg/h, Braun^®^, Barcelona, Spain). Additionally, meloxicam (0.2 mg/Kg/IV, Metacam, Boehringer Ingelheim España, Barcelona, Spain) was administered as a nonsteroidal anti-inflammatory analgesic [45]. Each animal received antibiotic prophylaxis with cefazolin (22 mg/Kg/IV, Cefazolina Normon^®^, Normon laboratories, Madrid, Spain), one dose 30 min prior to the surgery and another dose 60 min after the first incision.

The anesthesia was inducted with propofol injection (2 mg/Kg/IV, Propofol Lipuro^®^ 1%, Melsungen, Alemania). After endotracheal intubation, the patient was connected to an appropriate breathing system and the anesthesia was maintained with sevoflurane (vaporizer setting 2%) (SevoFlo^®^, Esteve Veterinary, Barcelona, Spain) in O_2_ 100%. The anesthetic plane was evaluated every 15 min by eye position, jaw tone, and palpebral reflex. F_E’_Sevo was adjusted accordingly by increasing or decreasing 0.5% of the previous vaporizer setting concentration each time until the appropriate anesthetic plane was achieved, which was established by the absence of palpebral reflex, loss of jaw tone, and ventrally rotated eye position [46,47]. If it was not enough, 1 mg/kg IV bolus of propofol was administered. Patients were placed in dorsal recumbency with a 15° head-up tilt (anti-Trendelenburg) and were mechanically ventilated in a volume-controlled mode with a ventilator (Mindray Anaesthesia Machine, WATO EX-65 Pro, Shenzhen, China). The initial tidal volume (TV) was established at 10 mL/Kg and afterwards was adjusted to maintain normocapnia, thereby establishing end-tidal carbon dioxide partial pressure (P_E’_CO_2_) at 35–45 mmHg. Additionally, we established a respiratory rate (RR) of 12–14/min, an inspiratory to expiratory ratio of 1:2, positive end-expiratory pressure (PEEP) of 4 cmH_2_O and inspiratory pause of 50% inspiratory time.

Additionally, prior to surgery, the dorsal metatarsal artery was percutaneously catheterized with a 22–24 gauge catheter for invasive blood pressure monitoring and arterial blood collection (blood gas and electrolyte studies). This catheter was removed before anesthesia recovery. For postoperative pain management, meloxicam (0.2 mg/kg/PO) was administered by owners for three days following the day of the surgical procedure [48,49]

### 2.4. Surgical Procedure

#### 2.4.1. Laparoscopic Ovariectomy Group

The LAP Ove surgeries were performed using a 3-portal technique with linear abdominal access [50]. The first trocar (6 mm) was placed 1 cm caudal to the umbilicus using the modified Hasson technique [51]. PNP was created with CO_2_ insufflation, establishing an IAP of 8–9 mmHg which was maintained throughout the surgery. The 5 mm optic was introduced, and the remaining trocars were placed under direct vision. The second trocar (11 mm with reducer) was placed at an intermediate distance between the pubis and the umbilicus, while the third trocar (6 mm) was introduced 1 cm cranial to the umbilicus.

After the placement of the access portals, a general abdominal exploration was performed, and the patient was placed into left lateral recumbency for removing the right ovary and subsequently into right lateral recumbency for removing the left ovary. The coagulation and cutting of both ovarian pedicles, suspensory ligament, and the uterine horn were performed using a 5 mm harmonic scalpel forceps. Once the absence of bleeding was verified, the two ovaries were removed through the caudal port (11 mm trocar). The portal sites were closed by a simple continuous layer in the abdominal fascia using a 0 absorbable monofilament glyconate suture (Monosyn, B. Braun VetCare SA, Barcelona, Spain) and a simple intradermal pattern on the skin with a 2-0/3-0 absorbable suture (Monosyn, B. Braun VetCare SA, Barcelona, Spain).

#### 2.4.2. Laparotomy Ovariectomy Group

For the conventional approach, an incision was made approximately 1 cm caudal of the umbilicus along the midline toward the pubis, for a length of approximately 4–8 cm (depending on the size of the patient) [10]. After the dissection of the subcutaneous tissue, the *linea alba* was located and a stab incision was made to access the abdominal cavity. Subsequently, a general inspection of the abdominal cavity was performed. Once the right ovary was located, a traction was carefully applied to allow its exteriorization and subsequent resection using the harmonic scalpel. Next, the left ovary was located and resected in the same way. After assessing the absence of bleeding, surgical closure was performed with 3 simple interrupted layers, using 0 monofilament suture in the abdominal fascia, 2-0 suture for subcutaneous layer, and a simple intradermal pattern on the skin with 2-0/3-0 absorbable suture.

### 2.5. Blood Sampling and Recorded Data

Anesthetic monitoring of the respiratory and hemodynamic variables was recorded using a multi-parametric monitor (Mindray multi-parametric monitor, iPM12 Vet). The parameters were manually collected every five minutes during the surgical procedure, but the statistical comparison of the data was performed with the values recorded at four different times: before PNP at the beginning of the surgery (T1), after the resection of the right ovary (T2), after the resection of the left ovary (T3), and at surgical closure after PNP deflation (T4). The following variables were considered for the study: the end-tidal CO_2_ (P_E’_CO_2_, mmHg), end-tidal concentration of sevoflurane (F_E’_Sevo; %), minute volume (MV; L/minute), peak inspiratory pressure (Ppeak; cmH_2_O), plateau pressure (Pplat; cmH_2_O), respiratory system compliance (C; mL/cmH_2_O), airway resistance (Raw; cmH_2_O), heart rate (HR; beats/minute), systolic (SAP; mmHg), diastolic (DAP; mmHg) and mean (MAP; mmHg) invasive blood pressure.

Additionally, blood samples were drawn before PNP insufflation (baseline) and immediately after deflation. Specifically, biochemical, and hematological parameters were determined by venous blood sampling (3 mL per sample): glucose (GLU; mg/dL), creatinine (CREA, mg/dL), total plasma proteins (TPP; g/dL), albumin (ALB; g/dL), globulins (GLOB; g/dL), alanine aminotransferase (ALT; UI/L), alkaline phosphatase (ALP; UI/L), hematocrit (PCV; %), hemoglobin concentration (HB; g/dL), white blood cells (WBC; K/μL), and platelet counts (PLT; K/μL). The biochemical parameters were carried out with an automatic biochemical analyzer (Catalyst One, IDEXX, Spain), and the hematologic values were obtained with the hematological analyzer (ProCyte Dx, IDEXX, Madrid, Spain). Moreover, arterial blood collection (1 mL per sample) was also performed using a heparinized syringe. Hydrogen ion concentration (pH), arterial partial pressure of carbon dioxide (PaCO_2_; mmHg), arterial partial pressure of oxygen (PaO_2_; mmHg)_,_ bicarbonate concentration (HCO_3_^−^; mEq/L), base excess (BE; mmol/L) and sodium, potassium, and chloride as electrolytes concentrations (Na^+^, K^+^, Cl^−^; mmol/L) (Electrolyte and Blood Gas Analyzer, IDEXX VetStat^*^, Madrid, Spain) were measured immediately after withdrawal.

### 2.6. Statistical Analysis

Statistical analyses were performed using the commercial software Sigma Plot 12.5 (Systat Software Inc., Chicago, IL, USA). The normality of the data was assessed using the Shapiro–Wilk test, and a Levene’s test was employed to assess the equality of variances of normal variables.

The analysis for the cardiorespiratory and F_E’_Sevo values, among the four different times (T1–T4), within the same group, were conducted using a repeated measures one-way analysis of variance (ANOVA) and by applying a *post-hoc* Holm–Sidak test. For non-normal variables, the statistical comparison was performed using repeated measures Friedman analysis followed by *post-hoc* Tukey test. Additionally, a paired Student’s *t*-test (normal distribution) or a Wilcoxon Signed Rank test (non-normal distribution) was used to compare the pre- and post- operative blood variables. The comparison of the variables between the two surgical groups (LAP Ove and LPT Ove group) was tested for significance using an unpaired Student’s *t*-test, or with a two-tailed Mann–Whitney U test when data did not follow a normal distribution. For all variables, the differences were considered statistically significant when *p* < 0.05.

## 3. Results

The procedures were performed between March 2019 and June 2019. Twenty client-owned dogs were initially enrolled in the study, but only sixteen healthy adult bitches were finally included. The four animals were excluded due to surgical or anesthetic complications and/or rescue analgesic requirements. All results are given as mean ± standard deviation (SD).

### 3.1. Age, Weight and Body Condition

The animals did not show any statistically significant differences in terms of weight, age, or body condition. The mean weight of the LAP Ove group was 26 ± 6.6 kg (ranging from 19 to 40 kg). In the LPT Ove group, the mean weight was 27.5 ± 8.6 (ranging from 17 to 45 kg). The mean age of the animals included in the LAP Ove group was 42 ± 30.5 months (ranging from 13 to 104 months), and in the LPT Ove group it was 40 ± 23 months (ranging from 13 to 72 months). As for mean body condition, it was normal with values of 2.9 ± 0.35 (ranging from 2 to 3) in the LAP Ove group and 2.6 ± 0.5 (ranging from 2 to 3) in the LPT Ove group.

### 3.2. Cardiorespiratory and End-Tidal Sevoflurane Concentration Values

**Figure 1:**
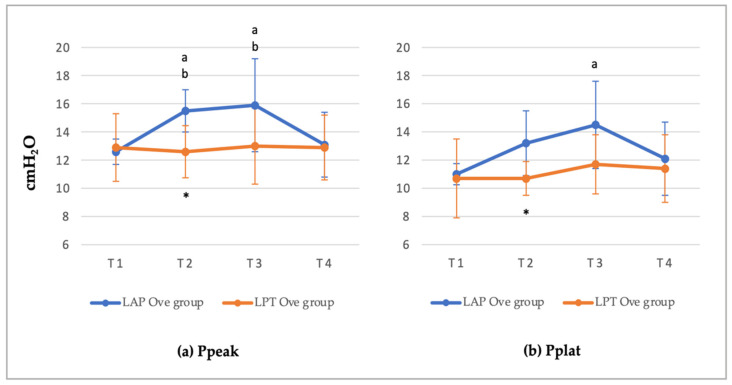
(**a**) Peak inspiratory pressure (Ppeak) and (**b**) Plateau pressure (Pplat) values determined at different points in time along the experimental period. Values given as a mean ± SD. *p* < 0.05: * vs. the LAP Ove group at the same time; ^a^ vs. T1 and ^b^ vs. T4 within the same Ove group.

All cardiorespiratory parameters and F_E’_Sevo values recorded are shown in Table S1. Among the ventilatory parameters recorded, no significant differences were observed in the values of P_E’_CO_2_, MV, or Raw in any of the groups studied, thus remaining within normal values at the four evaluation times. With regards to the respiratory pressure values recorded during mechanical ventilation, increased Ppeak and Pplat values were observed in the LAP Ove group during the establishment of PNP (T2 and T3), this difference being significant at T2 when compared to the LPT Ove group. In the case of Ppeak, the values recorded were 15.5 ± 1.5 cmH_2_O in the LAP Ove group versus 12.6 ± 1.85 cmH_2_O in the LPT Ove group, *p* = 0.04. As for Pplat, the values recorded were 13.2 ± 2.3 cmH_2_O were recorded in the LAP Ove group versus 10.7 ± 1.2 cmH_2_O in the LPT Ove group, *p* = 0.016. Analyzing the differences of pressure within the LAP Ove group, we determined statistically significant differences in Ppeak at T2 (15.5 ± 1.5) vs. T1 (12.6 ± 0.9, *p* = 0.008) and vs. T4 (13.1 ± 2.3, *p* = 0.002) and at T3 (15.9 ± 3.3) vs. T1 (12.6 ± 0.9, *p* = 0.003) and vs. T4 (13.1 ± 2.3, *p* = 0.009). Likewise, significant differences were observed in Pplat between T1 and T3 (11 ± 0.75 cmH_2_O vs. 14.5 ± 3.1 cmH_2_O, *p* = 0.007) (Figure 1).

**Figure 2:**
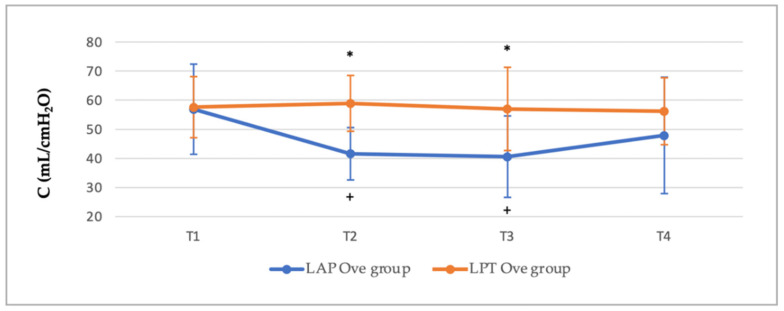
Compliance (C) values at different points in time along the experimental period. Values given as a mean ± SD. *p* < 0.05: * vs. the LAP Ove group at the same time; ^+^ vs. T1 within the same Ove group.

With regards to the compliance values, the lowest results were determined in the LAP Ove group during the T2 and T3 periods, simultaneously to the establishment of PNP. When comparing the results with those obtained in the LPT Ove group, significant differences were found at T2 (41.6 ± 9 mL/cmH_2_O in the LAP Ove group versus 58.9 ± 9.6 mL/cmH_2_O in the LPT Ove group, *p* = 0.002). Significant differences were also determined at T3 (40.6 ± 14 mL/cmH_2_O in the LAP Ove group versus 57 ± 14.3 mL/cm_2_H_2_O in the LPT group, *p* = 0.037). Likewise, when analyzing the differences within each group, statistical significances were determined in the LAP Ove group between T1 vs. T2 and T1 vs. T3 where there was a decrease from 56.9 ± 15.5 mL/cmH_2_O at T1 to 41.6 ± 9 mL/cmH_2_O at T2, *p* ≤ 0.001 and to 40.6 ± 14 at T3, *p* ≤ 0.001 (Figure 2).

**Figure 3:**
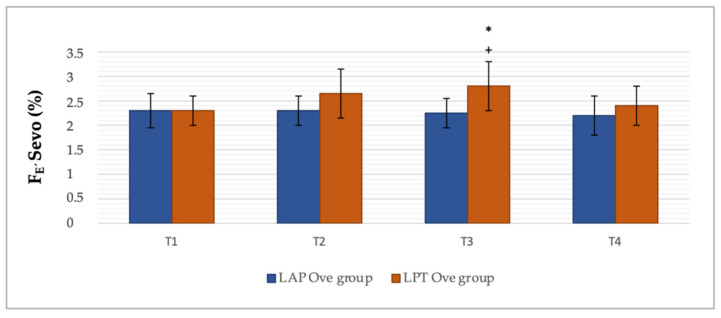
Percentage of end-tidal sevoflurane concentration (F_E’_Sevo) at different points in time along the experimental period. Values given as a mean ± SD. *p* < 0.05: * vs. the LAP Ove group at the same time; ^+^ vs. T1 within the same Ove group.

With regards to the F_E’_Sevo concentrations, higher values were recorded in the LPT Ove group, and they were statistically significant at T3 (after resection of the left ovary). In the LAP Ove group, the F_E’_Sevo at T3 was 2.25 ± 0.3 group compared to 2.8 ± 0.5 in the LPT Ove group, *p* = 0.019. Additionally, in the LPT Ove group, a significant increase in the F_E’_Sevo was observed between T1 and T3, determining an increase by 0.5% (2.3 ± 0.3 at T1 versus 2.8 ± 0.5 at T3, *p* = 0.0026) (Figure 3).

In terms of cardiovascular alterations, no significant differences were observed in HR between the studied groups nor at the evaluation times.

**Figure 4:**
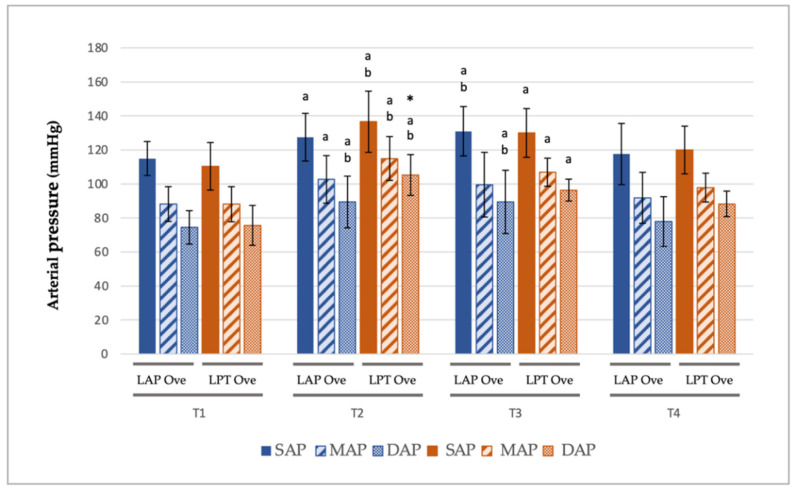
Invasive arterial blood pressure at different points in time along the experimental period. Values given as a mean ± SD. *p* < 0.05: * vs. the LAP Ove group at the same time; ^a^ vs. T1 and ^b^ vs. T4, within the same Ove group.

In the case of arterial pressure, in both groups significant higher-pressure values were recorded during ovarian resection (T2 and T3). This difference was especially marked at T2 in the LPT Ove group where the following data were recorded: an increase of 27 mmHg in SAP (110,4 ± 14.5 mmHg at T1 versus 136.6 ± 18 mmHg at T2, *p* ≤ 0.001), 29 mmHg in DAP (75.7 ± 11.7 mmHg at T1 versus 105.3 ± 12 mmHg at T2, *p* ≤ 0.001) and 27 mmHg in MAP (88.1 ± 10.3 mmHg at T1 versus 115 ± 12.9 mmHg at T2, *p* ≤ 0.001). Additionally, statistically significant differences were observed in DAP at T2 when comparing both groups (105.3 ± 12 in the LPT Ove group vs. 89.4 ± 15.2 in the LAP Ove group, *p* = 0.047) (Figure 4). 

### 3.3. Blood Gas and Hematochemical Analysis

Within the arterial gasometrical parameters determined (pH, PaCO_2_, HCO_3_, BE, and PaO_2_,) no statistically significant differences were observed in any of the groups evaluated, and all the parameters remained within the established reference values. Regarding electrolyte levels, a significant decrease in the Na^++^ values was determined in the LAP Ove group between pre- and postoperative analysis (154.35 ± 2.8 vs. 151.6 ± 31, *p* = 0.007). Significant differences were also observed in the pre-operative Na^++^ values analyzed between the LAP Ove and LPT Ove groups (154.25 ± 2.8 vs. 150.75 ± 3.3 mmol/L, *p* = 0.037), although all of these measurements were within the reference parameters.

Likewise, when K^+^ levels were evaluated, a statistically significant difference was determined in the LPT Ove group between pre- and postoperative assessment, increasing from 3.71 ± 0.2 to 4 ± 0.16 mmol/L, *p* = 0.032. Nevertheless, these values were also within the normal range. The results of the arterial blood analysis are shown in Table 1.

In relation to the results obtained in the biochemical and hematological studies (Table 2), a noticeable increase in the GLU concentration was observed after surgery in both groups. However, no statistically significant differences were observed between the two groups. In the LAP Ove group, there was an increase from 102.9 ± 18.5 to 151.2 ± 34.8 mg/dL, *p* = 0.003 and in the LPT Ove group from 107.87 ± 17.2 to 153.8 ± 29.3 mg/dL, *p* = 0.0019. Likewise, as for the TPP, ALB, and GLOB concentrations, a statistically significant decrease was determined after surgery in both surgical groups. After analyzing the other parameters such as CREA, ALT, and ALP, no significant differences were observed between the two treatment groups or between assessment times.

In the hematological study performed after surgery, no statistically significant differences were determined between the LAP and LPT Ove groups in the PCV, HB, and PLT values. However, there was a significant decrease in both groups between pre- and postoperative assessment. As for the WBC values analyzed, both groups showed a significant decrease in their values after surgery. Likewise, a statistical significance was established when comparing both groups, obtaining a difference of 7.35 ± 1.8 K/μL in the LAP Ove group versus 10.45 ± 2.4 K/μL in the LPT Ove group, *p* = 0.011.

## 4. Discussion

The laparoscopic OVE procedure has been determined as a valid and safe neutering method in dogs, being a less invasive and painful technique than the conventional procedure [18,38] However, it has also been associated with the occurrence of pathophysiological changes related to the establishment of PNP [17,21,24,41]. To the best of our knowledge, there is no study evaluating the cardiovascular and ventilatory parameters and complete blood analyses during the PNP in LAP Ove compared to conventional open ovariectomy in dogs. The same applies to MAC requirements of sevoflurane during anesthesia depending on the surgical technique.

As previously described, noticeable respiratory changes were found in laparoscopy surgeries mainly associated with the increase of IAP, which caused a cranial displacement of the diaphragm, decreasing the pulmonary distension, and reducing the functional residual capacity [17,19,20,52]. In our study, the establishment of PNP during the LAP Ove surgery resulted in a decrease in the compliance and an increase in the Ppeak and Pplat without affecting airway resistance. These results were in agreement with those observed by other authors, which determined that the establishment of the PNP, necessary to perform laparoscopic surgery, produced significant changes in pulmonary compliance and airway pressures [17,19,31,52]. For that reason, some authors have examined the use of spontaneous ventilation during laparoscopy surgeries [21,34] based on the fact that during mechanical ventilation, there is an increase in the inspiratory pressures. This pressure, in combination with an increased IAP, may in turn cause an increase in intrathoracic pressure thus reducing the cardiac output. Nevertheless, other studies associated spontaneous ventilation with a higher prevalence of respiratory problems, such as ventilation/perfusion disturbances and hypoventilation [17,20]. Additionally, similar to the results of our study, Raugh et al. [19] demonstrated that although the creation of PNP induced ventilatory changes, these quickly returned to normal values after deflation.

The P_E’_CO_2_ results were similar to those published by Lee & Kim [18], that is, no increased values during PNP were observed. The use of mechanical ventilation during LAP Ove seems to be capable of compensating the hypercapnia induced after CO_2_ absorption, maintaining the P_E’_CO_2_ values in normocapnia. A previous study on dogs [25] showed that in order to eliminate the absorbed CO_2_ through the peritoneal cavity, an increased minute ventilation was required. Similar to the results obtained by Shih et al. [27], our study did not find more requirements associated with the PNP. It may be associated with the use of low IAP values which should be better tolerated by the patients [22].

As for the F_E’_Sevo values registered, we observed that during ovarian resection, coinciding with a greater pain stimulus cause by the surgery, higher anesthetic requirements were determined in the LPT Ove group. This difference was statistically significant at T3 (left ovary resection), recording an increase by 0.55% of F_E’_Sevo with respect to the LAP Ove group at the same point in time. MAC is a reliable quantitative measurement of the potency of inhaled anesthetic agents [53]. Furthermore, ovarian stimulation pain is frequently noted in spaying procedures in companion animals [54]. In this regard, the increase in the anesthetic MAC requirements in conventional open neutering surgery was in line with other research studies, in which it was determined that laparoscopy techniques caused less pain and surgical stress than conventional ones due to the smaller incision required and less traction on the ovarian pedicle [1,3]. To the best of the authors´ knowledge, there are no current scientific studies in veterinary medicine that specifically focus on the MAC requirements of sevoflurane associated with the surgical ovariectomy technique in dogs. Therefore, we consider that further studies are required in order to examine these preliminary values in a more specific and exhaustive manner.

In the study of the hemodynamic response to PNP, it was described that these alterations were mainly observed at the beginning of the surgical procedure as a result from the increase of the IAP, the patient position, and the hypercapnia, which can result in a decreased cardiac output. Additionally, it is well-known that hemodynamic perturbations are pronounced in patients with cardiac and vascular disease [32]. At present, the prevention of the cardiovascular alterations is being thoroughly studied. In a recent publication, Di Bella et al. [33] have shown that the pulse pressure variation can be a valid predictor of the hemodynamic response to laparoscopy in dogs. Moreover, it could be used to prevent the cardiac output reduction by allowing to optimize the fluid therapy before PNP.

In our study, in the evaluation of the cardiovascular changes in healthy dogs, we determined the highest arterial blood pressure values during ovarian resection. Although hardly any significant differences were recorded between both surgical groups, this increase was slightly more marked at T2 in the LPT Ove group compared to the LAP Ove group. This is in line with the findings of other authors, and it was probably associated with a higher pain stimulus [1,41]. Regarding pain management during ovarian ligation, in a recent publication Cicirelli et al. [7] have investigated the potential benefits of using local anesthetic drugs in order to provide an adequate analgesia. The study showed that a local splash block using lidocaine resulted in greater hemodynamic stability and lower surgical pain. Likewise, increases in the arterial pressure were also recorded during trocar placement. It has been demonstrated that the use of a smaller number of portals [2,41,55,56] and smaller instruments [57] could be beneficial in contributing to decreased intraoperative pain. Regarding HR, tachycardia was described in laparoscopic surgery as a compensatory response related to the peritoneal cavity distension, as well as the stimulatory effects of hypercapnia [24]. Nevertheless, it seems that the use of lower IAP helps to prevent significant cardiovascular changes [25,26]. In our case, in line with other studies [10,11,18,41,52], no significant variations in the HR were determined. This was probably due to the PNP values established (8–9 mmHg), the type of premedication and anesthesia, as well as a possible baroreceptor reflex caused by the increased arterial pressure.

As we previously mentioned, among the pathophysiological changes associated with the establishment of PNP, in addition to the alterations caused by the IAP mechanical effect, metabolic and respiratory changes were also observed as related to the absorption of CO_2_ through the peritoneum [16,20,21,25,27,37]. Among the main changes described, there were increases in the PaCO_2_, decreases in the pH, decreases in the PaO_2_ values, and increases in the plasma–bicarbonate concentrations. In our study, after evaluating the main pre- and post-PNP arterial blood gasometrical parameters, we did not determine statistically significant variations depending on the surgical technique or blood sample collection time. This was similar to other studies where the patient was mechanically ventilated during laparoscopic surgery [17,18]. Therefore, the use of mechanical ventilation at positive low volumes, and with the application of PEEP, seems to be crucial to ensure an effective ventilation, preventing alterations in the gas exchange rate, the appearance of atelectasis, and improving the lung function and respiratory mechanics [17,20]. Additionally, Russo et al. also observed an improved cardiac function associated with an enhanced CO_2_ washout, oxygenation, and a vasoconstriction inhibition [30].

Regarding the biochemical analyses performed, in the evaluation of the GLU plasma concentrations, in line with other publications [11,38], significantly higher values were determined in both groups after surgery. However, no statistically significant differences were observed depending on the surgical technique used. On the contrary, Devitt et al. [1] recorded higher and prolonged hyperglycemia in the open OHE group compared to the laparoscopic-assisted one. Nevertheless, we should take into account that although blood GLU concentration has been examined in small animals as an indicator of postoperative pain [58] and surgical stress response [59], some authors did not determine this parameter as a useful measure of surgical stress or pain [11]. In relation to the ALB, TPP, and GLOB levels, decreased values were determined after surgery in both groups. This is different from what was observed in another publication [40], in which no significant differences were determined in the serum ALB values among the sampling times.

As for the blood count parameters such as PCV, HB and PLT, there was a significant decrease after surgery in both groups, probably due to perioperative fluid administration, splenic sequestration, and surgical trauma. These results were in line with those published recently by Rubio et al. [60], where significantly decreased values were also determined in PCV and PLT compared to baseline after open OHE in healthy dogs. Additionally, some authors observed greater loss of blood in the laparotomy with respect to laparoscopic techniques [10,18]. Shariati et al. [10] determined less hemorrhage in LAP Ove than in the conventional approach. Nevertheless, similar to the results of our study, they did not find any differences in the complete blood count parameters between both groups. As for the concentration of WBC, previous publications have reported significantly increased post-operative values after OHE [42,60]. However, in our study, both groups showed a significant decrease in the immediately postoperative period, probably due to hemodilution. Nevertheless, this descent was more noticeable in the LAP Ove animals. The slightly higher leukocytes values observed in the LPT Ove group could correspond to a greater inflammatory and immune response, which is determined by a more invasive and painful technique than that performed with laparoscopic surgery [1].

Finally, a better understanding of the pathophysiological effects of PNP is crucial for appropriate anesthetic and surgical planning. This is especially important in prolonged procedures or in high-risk patients with previous cardiorespiratory and metabolic pathologies. Although the insufflation of the abdominal cavity with CO_2_ has been associated with numerous physiological and pathological changes, it has been proven that the use of low IAP [22], in addition to an adequate selection of the parameters in the positive pressure ventilation, could allow the patients to physiologically respond, thus reducing the perioperative complications [20,30]. In conclusion, the results of our study suggested that local and systemic alterations related to PNP in LAP Ove seemed to be reversible after CO_2_ deflation and well-tolerated by healthy patients. Thus, the laparoscopic approach for Ove should increasingly be considered the “gold standard” in neutering procedures in the veterinary field due to their lower invasiveness, better visualization, and fewer complications.

This study had several limitations, mainly that of a small patient population (limited to 8 dogs per group). As such, the results should be interpreted with caution. Therefore, further studies, including a power calculation, may be necessary to corroborate our preliminary results. Additionally, more blood sample collections during the surgery procedure and some hours afterwards would have contributed to a more complete evaluation of the pathophysiological changes under study. Lastly, taking into account the variety of LAP Ove techniques described in the literature, with a different number of portals described, the use of two portals rather than three may have been more appropriate, as recommended by some authors [2,41,55].

## 5. Conclusions

The establishment of PNP in LAP Ove results in significant ventilatory changes with respect to open conventional surgery, determining a reduction in the pulmonary compliance and increased inspiratory pressures. However, the use of mechanical ventilation at low volumes, with the application of PEEP, may provide an effective ventilation and compensate the hypercapnia induced after the absorption of CO_2_ through the abdominal cavity. No variations were recorded in the gasometrical parameters of any of the groups studied. In the study of the MAC requirements of sevoflurane, we determined significantly higher percentages of F_E’_Sevo concentrations in the LPT Ove group during ovarian resection, coinciding with the maximum values of arterial pressure recorded, and being determined as the greatest intraoperative pain stimulus. Lastly, in relation to the hematochemical parameters, no significant differences were determined depending on the surgical technique. However, although all the values remained within normal ranges, some of the parameters evaluated in the postoperative blood sample collection could indicate a higher surgical stress in the open surgery group with respect to the minimally invasive technique. Thus, these findings may support the numerous advantages of minimally invasive surgery compared to open surgery for neutering female dogs.

## Figures and Tables

**Table 1:** Pre- and postoperative arterial blood analysis.

	LAP Ove Group		LPT Ove Group	
Variables	PRE	POST	PRE	POST
pH	7.38 ± 0.05	7.39 ± 0.05	7.40 ± 0.04	7.41 ± 0.04
PaCO_2_ (mmHg)	41.25 ± 3	40.12 ± 4.3	41.9 ± 2.35	39.12 ± 4
HCO_3_ (mEq/L)	24.28 ± 1.7	23.1 ± 1.29	25.4 ± 1.34	24.75 ± 1.24
BE (mmol/L)	−0.38 ± 1.8	−1.6 ± 1.4	1.07 ± 1.44	0.46 ± 1.3
PaO_2_ (mmHg)	418 ± 58	434 ± 32	434 ± 40	429 ± 45
Na^++^ (mmol/L)	154.25 ± 2.8	**151.6 ± 3.1 ^+^**	**150.75 ± 3.3** *	150.5 ± 1.8
K^+^ (mmol/L)	3.62 ± 0.4	3.99 ± 0.6	3.71 ± 0.2	**4 ± 0.16 ^+^**
Cl^−^ (mmol/L)	119.25 ± 1.99	119 ± 1.5	117.62 ± 1.5	118 ± 1

Arterial blood data. Values given as a mean ± SD. Statistically significant differences are marked in “bold text”. *p* < 0.05: * vs. the LAP Ove group at the same time; ^+^ vs. pre-operative values within the same group.

**Table 2:** Pre- and postoperative biochemical and hematologic analysis.

	LAP Ove Group		LPT Ove Group	
Variables	PRE	POST	PRE	POST
GLU (mg/dL)	102.9 ± 18.5	**151.2 ± 34.8 ^+^**	107.87 ± 17.2	**153.8 ± 29.3 ^+^**
CREA (mg/dL)	0.85 ± 0.14	0.85 ± 0.14	0.7 ± 0.15	0.71 ± 0.13
TPP (g/dL)	6.7 ± 0.6	**6.1 ± 0.8 ^+^**	6.5 ± 0.4	**6 ± 0.5 ^+^**
ALB (g/dL)	3.2 ± 0.16	**2.8 ± 0.2 ^+^**	2.9 ± 0.2	**2.7 ± 0.2 ^+^**
GLOB (g/dL)	3.57 ± 0.6	**3.26 ± 0.6 ^+^**	3.59 ± 0.35	**3.3 ± 0.3 ^+^**
ALT (UI/L)	39.37 ± 18.4	37.37 ± 22.2	34.6 ± 12.4	30.87 ± 13.5
ALP (UI/L)	49.87 ± 21.16	44.75 ± 20.1	45 ± 23.5	37 ± 19.6
PCV (%)	43.3 ± 7.3	**39.7 ± 6.6 ^+^**	43.3 ± 3.9	**38.1 ± 3.8 ^+^**
HB (g/dL)	15.82 ± 2.6	**14.56 ± 2.5 ^+^**	15.9 ± 1.16	**14 ± 1.34 ^+^**
WBC (K/μL)	11.34 ± 3.2	**7.35 ± 1.8 ^+^**	13.9 ± 2.38	**10.45 ± 2.4** *^, +^
PLT (K/μL)	327.5 ± 127	**293.4 ± 98.8 ^+^**	324.5 ± 29.7	**283.5 ± 33.2 ^+^**

Venous blood data. Values given as mean ± SD. Statistically significant differences are marked in “bold text”. *p* < 0.05: * vs. the LAP Ove group at the same time; ^+^ vs. pre-operative values within the same group.

## Data Availability

Not applicable.

## Supplementary Materials

The following supporting information can be downloaded at: https://www.mdpi.com/article/10.3390/ani12111438/s1, Table S1: Cardiorespiratory values and MAC sevoflurane requirements.
