# Supplementary material for: Laparoscopic versus Open Ovariectomy in Bitches: Changes in Cardiorespiratory Values, Blood Parameters, and Sevoflurane Requirements Associated with the Surgical Technique
Source: Animals (Basel). 2022 Jun 2;12(11):1438. doi: 10.3390/ani12111438 (PMC9179476; doi:10.3390/ani12111438)
Supplement: Supplementary file 1 [file animals-12-01438-s001.zip › animals-1738557-supplementary.pdf]

Table S1. End-tidal sevoflurane concentration and cardiorespiratory parameters.

	LAP Ove group				LPT Ove group			
**Variables**	**T1**	**T2**	**T3**	**T4**	**T1**	**T2**	**T3**	**T4**
**$F_{E'}$Sevo (%)**	2.3 ± 0.35	2.3 ± 0.3	2.25 ± 0.3	2.2 ± 0.4	2.3 ± 0.3	2.65 ± 0.5	**2.8 ± 0.5** [*, a]	2.4 ± 0.4
**Ppeak (cmH_2O)**	12.6 ± 0.9	**15.5 ± 1.5** [a, b]	**15.9 ± 3.3** [a, b]	13.1 ± 2.3	12.9 ± 2.4	**12.6 ± 1.85** [*]	13 ± 2.7	12.9 ± 2.3
**Pplat (cmH_2O)**	11 ± 0.75	13.2 ± 2.3	**14.5 ± 3.1** [a]	12.1 ± 2.6	10.7 ± 2.8	**10.7 ± 1.2** [*]	11.7 ± 2.1	11.4 ± 2.4
**$P_{E'}CO_2$ (mmHg)**	41.4 ± 2.7	40.6 ± 1.4	42 ± 2.6	41.2 ± 2.7	41.2 ± 2.9	39.1 ± 2.1	39.2 ± 2.6	41.2 ± 2.5
**MV (L/min)**	5.4 ± 1.9	5.2 ± 1.3	5.5 ± 1.8	5.5 ± 1.5	5.7 ± 1.7	5.5 ± 1.6	5.8 ± 1.7	5.7 ± 1.7
**C (mL/cmH_2O)**	56.9 ± 15.5	**41.6 ± 9** [a]	**40.6 ± 14** [a]	47.9 ± 20	57.6 ± 10.5	**58.9 ± 9.6** [*]	**57 ± 14.3** [*]	56.2 ± 11.5
**Raw (cmH_2O)**	10.1 ± 1.2	11.9 ± 1.3	11.6 ± 3.1	12.1 ± 9.7	9.7 ± 2.5	10.4 ± 1.6	10.9 ± 3.3	10.2 ± 1.5
**HR (beats/min)**	84 ± 11.9	84.9 ± 13.8	85.9 ± 21.3	85.2 ± 16	84 ± 13.8	84.1 ± 19	84.8 ±18	86.8 ± 16.9
**SAP (mmHg)**	115 ± 10	**127.5 ± 14** [a]	**131 ± 14.5** [a, b]	117.6 ± 18	110.4 ± 14	**136.6 ± 18** [a, b]	**130 ± 14.3** [a]	120 ± 14
**DAP (mmHg)**	74.5 ± 9.8	**89.4 ± 15.2** [a, b]	**89.5 ± 18.6** [a, b]	77.9 ± 14.6	75.7 ± 11.7	**105.3 ± 12** [*, a, b]	**96.4 ± 6.4** [a]	88.3 ± 7.5
**MAP (mmHg)**	88.2 ± 10.2	**102.7 ± 14** [a]	99.6 ± 19	91.9 ± 15	88.1 ± 10.3	**115 ± 12.9** [a, b]	**106.9 ± 8.3** [a]	97.9 ± 8.5

Parameters evaluated in 16 bitches submitted to LAP Ove and LPT Ove. Values given as a mean ± SD. Statistically significant differences are marked in "bold text". $p < 0.05$: [*] versus the LAP Ove group at the same time; [a] vs. T1 and, [b] vs. T4 within the same group.
